# Hemophagocytic Lymphohistiocytosis in Association With *Clostridium difficile* Infection and Cutaneous T-Cell Lymphoma

**DOI:** 10.4021/wjon307w

**Published:** 2011-06-08

**Authors:** Suresh Kumar Nayudu, Nadia Fida, Anna Acidera, Myrta Daniel, Donald Rudikoff, Masooma Niazi, Sridhar Chilimuri

**Affiliations:** a Department of Medicine, Bronx Lebanon Hospital Center, Affiliated With Albert Einstein College of Medicine, Bronx, New York, USA; b Department of Pathology, Bronx Lebanon Hospital Center, Affiliated With Albert Einstein College of Medicine, Bronx, New York, USA

**Keywords:** Hemophagocytic lymphohistiocytosis, Clostridium difficile

## Abstract

Hemophagocytic lymphohistiocytosis (HLH) or Macrophage Activation Syndrome (MAS) is a potentially life threatening disorder that presents with fever, suppressed blood cell counts, hepatosplenomegaly and multi-organ failure. HLH has been reported in association with genetic mutations, infections, autoimmune disorders, and various malignancies. However to the best of our knowledge, HLH in association with *Clostridium difficile* infection has never been reported. We present a case of HLH in a patient with Epstein-Barr virus (EBV) positive natural killer T (NKT) cell cutaneous lymphoma and active *Clostridium difficile* infection. A 35-year-old male with recently diagnosed EBV positive NKT cell lymphoma was admitted for *Clostridium difficile* associated diarrhea. During the course of hospitalization he gradually developed pancytopenia and multi-organ failure leading to death. Post-mortem examination confirmed the diagnosis of hemophagocytic lymphohistiocytosis.

## Introduction

Hemophagocytic lymphohistiocytosis (HLH), also known as Macrophage Activation Syndrome (MAS) is potentially a life threatening disorder [1-3]. HLH clinically presents with fever, hepatosplenomegaly, suppressed blood cell counts, altered coagulation cascade and multi-organ dysfunction [2]. This syndrome has been reported frequently in infants and children compared to adults [4-6]. Infectious agents, autoimmune disorders and malignancies have been associated with HLH in adults [7, 8] whereas genetic mutations play a major role in infants and children [9-12]. Cutaneous T-cell lymphomas in the setting of latent Epstein-Barr virus (EBV) infection have been reported in the literature [13, 14]. However to the best of our knowledge natural killer T (NKT) cell lymphoma in presence of latent EBV and active *Clostridium difficile* infection, progressing to HLH has never been reported. We present a case of HLH in a patient with EBV positive NKT cell cutaneous lymphoma admitted with *Clostridium difficile* associated diarrhea.

## Case Report

**Figure 1:**
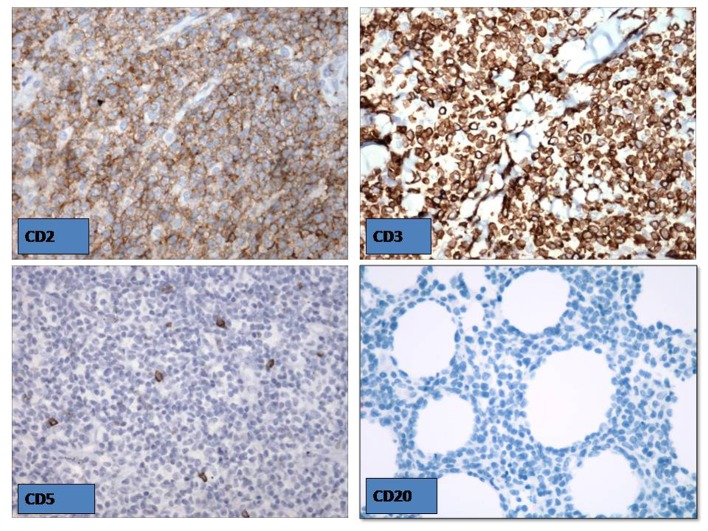
The skin biopsy revealed EBV positive NKT cell cutaneous lymphoma.

**Figure 2:**
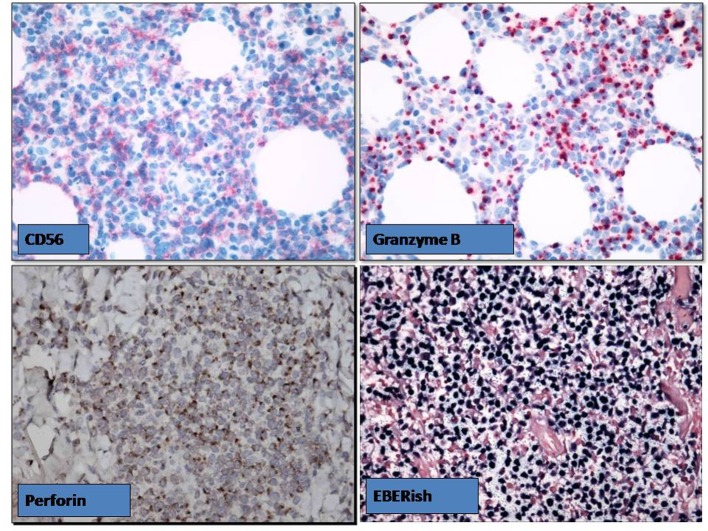
The skin biopsy revealed EBV positive NKT cell cutaneous lymphoma.

A 35-year-old Hispanic male presented to the emergency room with severe right sided abdominal pain of one week duration. The pain was 10/10 in intensity, non-radiating and associated with loss of appetite, nausea, vomiting and two to three episodes of watery diarrhea per day. There was no mucus or blood in stool. He also had subjective fever and denied any contact with sick people or recent travel. He denied any other medical problems. He had Bell’s palsy one month back which was treated with Valacyclovir and Prednisone. He had skin rash of three months duration associated with itching for which he underwent skin biopsy one week prior to admission at a different hospital. The biopsy was reported as EBV positive NKT cell cutaneous lymphoma (Fig. 1, 2). On further work up by his dermatologist his routine labs were within normal limits. He was also tested negative for Human Immunodeficiency Virus (HIV), Human T cell Lymphoma Virus (HTLV) I and HTLV II.

Review of other systems was non-contributory. He denied any family history of malignancies. He was an immigrant from Dominican Republic. He was an active smoker and drank alcohol occasionally but denied recreational substance use.

**Figure 3:**
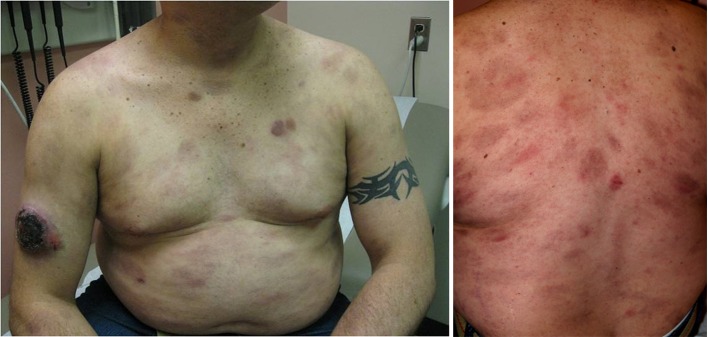
Diffuse erythematous violaceous rashes were presented on back, chest, abdomen, neck, and extremities.

**Figure 4:**
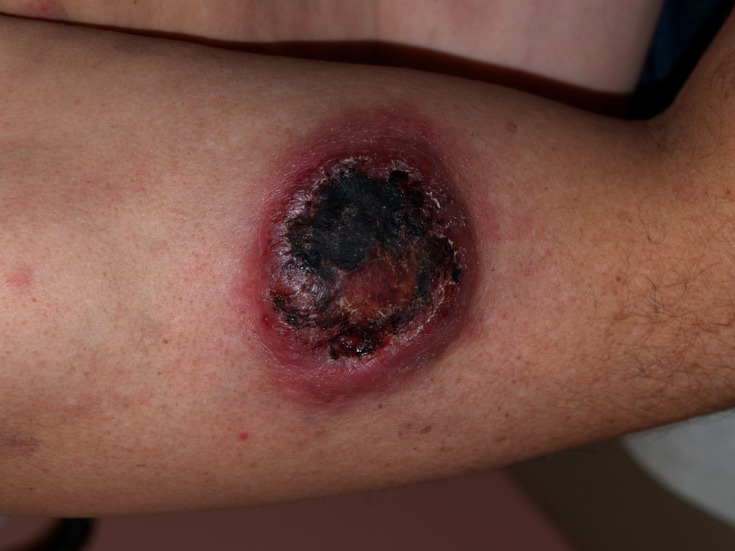
An ulcerated crusted plaque of 8 cm covered by eschar was noted on right arm.

He was afebrile and hemo-dynamically stable on presentation. Physical examination revealed diffuse erythematous violaceous rash on back, chest, abdomen, neck, and extremities (Fig. 3). An ulcerated crusted plaque of 8 cm covered by eschar was noted on right arm (Fig. 4). Abdomen was tender on right upper and lower quadrants with normoactive bowel sounds. Laboratory results showed white cell count of 4600/microliter, platelet count of 158,000/microliter. His basic metabolic panel, liver function tests, amylase and lipase levels were within normal limits. Computer Tomography (CT) of the abdomen showed benign angiomyolipoma of right adrenal gland and bilateral inguinal lymphadenopathy.

He was admitted and treated with intravenous (IV) fluids, metoclopramide and analgesics. On the second day of hospital course he had fever, so blood, urine and stool cultures were sent as a part of septic work up. His stool specimen was reported positive for *Clostridium difficile* toxin. He was treated with Vancomycin, Piperacillin + tazobactam and Metronidazole. He underwent esophagogastroduodenoscopy (EGD) due to intractable vomiting which showed hiatal hernia, and biopsy revealed minimally active gastritis, with negative test for *Helicobacter pylori*.

He has been febrile throughout his hospital course, and his liver enzyme levels were found to be elevated on 8th hospital day along with leucopenia and thrombocytopenia. His hepatitis viral titers were negative and there was no evidence of cholelithiasis on ultrasonogram. He underwent trans-thoracic echocardiogram and no vegetations were detected. His fever persisted for next several days and his blood cultures were negative repeatedly. He underwent biopsy of the left inguinal lymph node and pathology report was awaited. On 20th day of hospital course he was delirious and hypotensive with respiratory distress. He was intubated, started on vasopressors, Imipenem, Fluconazole, and Vancomycin; and transferred to critical care unit immediately. He did not improve and died on 22nd day of hospitalization.

**Figure 5:**
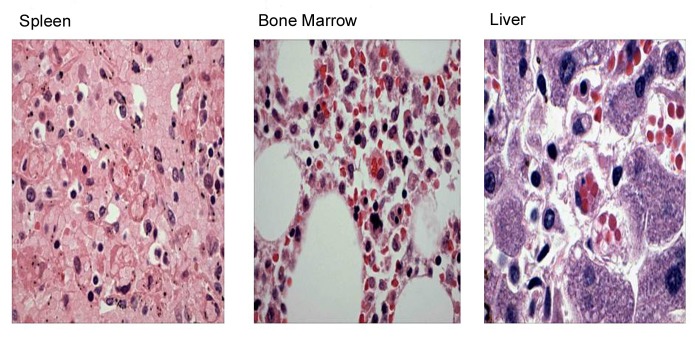
Autopsy results revealed hemophagocytic lymphohistiocytosis in bone marrow, liver and spleen.

His blood cultures from 20th day were growing *Enterococcus fecalis* and *Klebsiella pneumoniae* sensitive to Vancomycin and Imepenem respectively. Lymph node biopsy result was pending and autopsy was requested. Autopsy results revealed EBV positive cutaneous NKT cell lymphoma and secondary hemophagocytic lymphohistiocytosis in bone marrow, liver and spleen (Fig. 5).

## Discussion

Hemophagocytic lymphohistiocytosis is an inflammatory disorder [6] reported frequently in children [3, 5, 15, 16]. Familial HLH has higher prevalence in infants and children where genetic mutations play a role in the pathogenesis [6, 10-12, 17, 18]. Secondary HLH is seen in adults more frequently where autoimmune disorders [1, 8], malignancies [4, 19, 20], infectious agents including bacteria [21, 22], viruses [23, 24], and immunosuppression [7] are involved in the pathogenesis. Macrophage activation syndrome (MAS) is a variant of HLH especially when it occurs in association with autoimmune disorders [1, 2]. Clinical presentation may include fever, jaundice, hepatosplenomegaly, suppressed blood cell counts, altered coagulation profile and multi organ dysfunction [14] leading to death. Laboratory findings may include pancytopenia, abnormal liver function tests and altered coagulation profile [6, 14]. Some patients may have abnormal natural killer (NK) cell assay, elevated levels of ferritin and triglycerides [6, 17, 24, 25].

HLH has poor prognosis especially when it occurs in the presence of EBV infection and cutaneous NKT cell lymphomas [13, 14, 19, 23, 26, 27]. This case explains the complexity and poor prognosis associated with HLH. In this context patient had recently diagnosed EBV positive NKT cell lymphoma and was admitted with *Clostridium difficile* associated diarrhea. However there was no evidence of antibiotic usage prior to the presentation. Hence *Clostridium difficile* associated diarrhea in this case might be a community acquired infection [28]. Succeeding hepatic failure, pancytopenia, and post-mortem findings further confirm the diagnosis of HLH. We can argue that bacterial sepsis is a precipitating event of HLH, but initial negative blood cultures may support sepsis as a terminal event rather than precipitating event.

This patient had classic symptom and signs of HLH which include persistent fever, lymphadenopathy, elevated liver enzymes and multi-organ failure in the presence of active malignancy. However HLH precipitated in EBV positive NKT cell lymphoma patient by *Clostridium difficile* associated diarrhea is unique to this patient and has never been reported in the past. This case warrants us to consider HLH as a differential diagnosis whenever patients with active malignancy and metabolic stress like infection, present with clinical and laboratory findings suggestive of HLH. Since diagnosing at earlier stage may be helpful as present treatment regimens are proven to be having survival benefits [3, 29] physicians should be aware of early detection and appropriate management of HLH.

## References

[1] Tristano AG (2008). Macrophage activation syndrome: a frequent but under-diagnosed complication associated with rheumatic diseases. Med Sci Monit.

[2] Deane S, Selmi C, Teuber SS, Gershwin ME (2010). Macrophage activation syndrome in autoimmune disease. Int Arch Allergy Immunol.

[3] Ariffin H, Lum SH, Cheok SA, Shekhar K, Ariffin WA, Chan LL, Lin HP (2005). Haemophagocytic lymphohistiocytosis in Malaysian children. J Paediatr Child Health.

[4] Devecioglu O, Anak S, Atay D, Aktan P, Devecioglu E, Ozalp B, Saribeyoglu E (2009). Pediatric acute lymphoblastic leukemia complicated by secondary hemophagocytic lymphohistiocytosis. Pediatr Blood Cancer.

[5] Ishii E, Ohga S, Imashuku S, Yasukawa M, Tsuda H, Miura I, Yamamoto K (2007). Nationwide survey of hemophagocytic lymphohistiocytosis in Japan. Int J Hematol.

[6] Janka G, zur Stadt U (2005). Familial and acquired hemophagocytic lymphohistiocytosis. Hematology Am Soc Hematol Educ Program.

[7] Nogueira MV, Vidal L, Terra B, Pagot T, Salluh JI, Soares M (2009). Hemophagocytic syndrome associated with cytomegalovirus infection in a severely immunocompromised AIDS patient: case report. Braz J Infect Dis.

[8] Dierick M, Lacquet F, Verhelst C, Vonck A, Van Garsse L (2009). [Systemic lupus erythematosus over hemophagocytic lymphohistiocytosis]. Acta Clin Belg.

[9] Marsh RA, Satake N, Biroschak J, Jacobs T, Johnson J, Jordan MB, Bleesing JJ (2010). STX11 mutations and clinical phenotypes of familial hemophagocytic lymphohistiocytosis in North America. Pediatr Blood Cancer.

[10] Jakovljevic G, Kardum-Skelin I, Rogosic S, Culic S, Stepan J, Gagro A, Skaric I (2010). Familial hemophagocytic lymphohistiocytosis in a 6-week-old male infant. Coll Antropol.

[11] zur Stadt U, Rohr J, Seifert W, Koch F, Grieve S, Pagel J, Strauss J (2009). Familial hemophagocytic lymphohistiocytosis type 5 (FHL-5) is caused by mutations in Munc18-2 and impaired binding to syntaxin 11. Am J Hum Genet.

[12] Lu G, Xie ZD, Shen KL, Ye LJ, Wu RH, Liu CY, Jin YK (2009). Mutations in the perforin gene in children with hemophagocytic lymphohistiocytosis. Chin Med J (Engl).

[13] Smith KJ, Skelton HG, Giblin WL, James WD (1991). Cutaneous lesions of hemophagocytic syndrome in a patient with T-cell lymphoma and active Epstein-Barr infection. J Am Acad Dermatol.

[14] Su IJ, Hsu YH, Lin MT, Cheng AL, Wang CH, Weiss LM (1993). Epstein-Barr virus-containing T-cell lymphoma presents with hemophagocytic syndrome mimicking malignant histiocytosis. Cancer.

[15] Gurgey A, Unal S, Okur H, Orhan D, Yurdakok M (2008). Neonatal primary hemophagocytic lymphohistiocytosis in Turkish children. J Pediatr Hematol Oncol.

[16] Chan JS, Shing MM, Lee V, Li CK, Yuen P (2008). Haemophagocytic lymphohistiocytosis in Hong Kong children. Hong Kong Med J.

[17] Janka GE (2007). Familial and acquired hemophagocytic lymphohistiocytosis. Eur J Pediatr.

[18] Schultz KA, Neglia JP, Smith AR, Ochs HD, Torgerson TR, Kumar A (2008). Familial hemophagocytic lymphohistiocytosis in two brothers with X-linked agammaglobulinemia. Pediatr Blood Cancer.

[19] Romero LS, Goltz RW, Nagi C, Shin SS, Ho AD (1996). Subcutaneous T-cell lymphoma with associated hemophagocytic syndrome and terminal leukemic transformation. J Am Acad Dermatol.

[20] Sovinz P, Lackner H, Urban C (2008). Recurrent episodes of fever and pancytopenia due to haemophagocytosis during maintenance therapy for acute myeloid leukaemia. Br J Haematol.

[21] Balkis MM, Bazzi L, Taher A, Salem Z, Uthman I, Kanj N, Boulos FI (2009). Severe hemophagocytic syndrome developing after treatment initiation for disseminated Mycobacterium tuberculosis: case report and literature review. Scand J Infect Dis.

[22] Su NW, Chen CK, Chen GS, Hsieh RK, Chang MC (2009). A case of tuberculosis-induced hemophagocytic lymphohistiocytosis in a patient under hemodialysis. Int J Hematol.

[23] Iwatsuki K, Harada H, Ohtsuka M, Han G, Kaneko F (1997). Latent Epstein-Barr virus infection is frequently detected in subcutaneous lymphoma associated with hemophagocytosis but not in nonfatal cytophagic histiocytic panniculitis. Arch Dermatol.

[24] Rouphael NG, Talati NJ, Vaughan C, Cunningham K, Moreira R, Gould C (2007). Infections associated with haemophagocytic syndrome. Lancet Infect Dis.

[25] Allen CE, Yu X, Kozinetz CA, McClain KL (2008). Highly elevated ferritin levels and the diagnosis of hemophagocytic lymphohistiocytosis. Pediatr Blood Cancer.

[26] Iwatsuki K, Ohtsuka M, Harada H, Han G, Kaneko F (1997). Clinicopathologic manifestations of Epstein-Barr virus-associated cutaneous lymphoproliferative disorders. Arch Dermatol.

[27] Abe Y, Muta K, Ohshima K, Yasumoto S, Shiratsuchi M, Saito R, Tsujita J (2000). Subcutaneous panniculitis by Epstein-Barr virus-infected natural killer (NK) cell proliferation terminating in aggressive subcutaneous NK cell lymphoma. Am J Hematol.

[28] Laing RB, Dykhuizen RS, Smith CC, Gould IW, Reid TM (1996). Community-acquired toxigenic Clostridium difficile diarrhoea in the normoxaemic elderly who have received no antimicrobials: soft evidence for ischaemic colitis?. Scott Med J.

[29] Hasegawa D, Sano K, Kosaka Y, Hayakawa A, Nakamura H (1999). A case of hemophagocytic lymphohistiocytosis with prolonged remission after syngeneic bone marrow transplantation. Bone Marrow Transplant.

